# Rational design of efficient transition metal core–shell electrocatalysts for oxygen reduction and evolution reactions†

**DOI:** 10.1039/c8ra09122f

**Published:** 2019-01-02

**Authors:** Zhenghang Zhao, Jason D'Souza, Fuyi Chen, Zhenhai Xia

**Affiliations:** Department of Materials Science and Engineering, University of North Texas Denton TX 76201 USA Zhenhai.Xia@unt.edu; School of Materials Science and Engineering, Northwestern Polytechnical University Xi'an 710072 China

## Abstract

Ag can form core–shell structures with other non-precious transition metals, which is a promising candidate as an efficient and cost-effective electrocatalyst to replace Pt and RuO_2_ for oxygen reduction and evolution reactions (ORR and OER) in fuel cells and metal–air batteries. In this paper, polyicosahedral (plh) Ag_32_X_6_ (X = 3d transition metals) core–shell structures are calculated systematically by the density functional theory (DFT) method to predict their electrocatalytic activities for ORR and OER. It is found that the strain on the outer shell of the core–shell structures can be an intrinsic descriptor that describes the bifunctional catalytic activities of the catalysts. A higher compressive strain leads to more positive charge on the surface of the shell and consequently higher catalytic activities. The results provide a theoretical base for the rational design and screening of the Ag-based core–shell catalysts for clean energy conversion and storage.

## Abstract 2

Ag core–shell nanoparticle is a promising candidate as an efficient and cost-effective electrocatalyst for ORR and OER in fuel cells and metal–air batteries. Shell strain is the intrinsic descriptor to rationalize the electrochemical activity.

## Graphical abstract

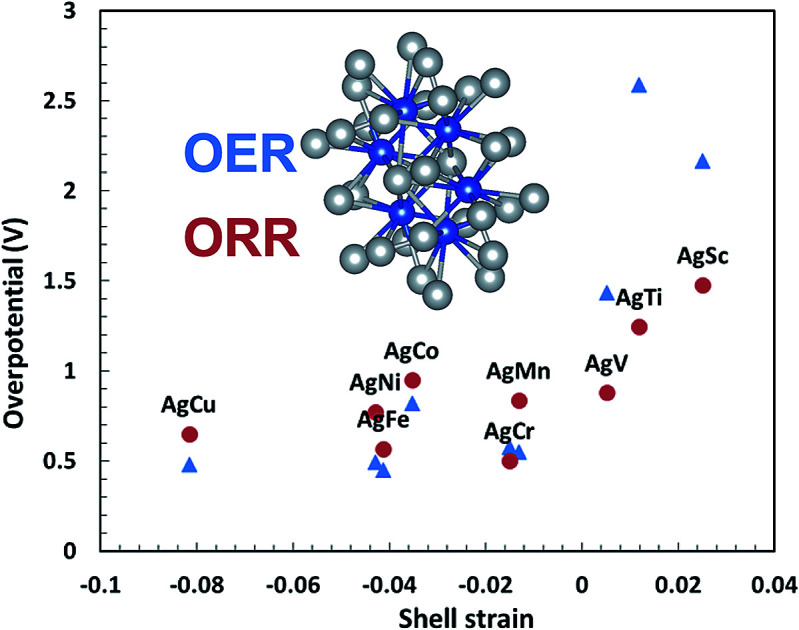

## Introduction

Fuel cells along with metal–air batteries are considered as next-generation clean energy technologies with numerous advantages such as being low cost, environment friendly, and easy to mass produce. These energy technologies can provide an optimal solution to clean and sustainable energy in combination with never-ending but intermittent natural sources such as wind power and solar energy.^1^ At the heart of the energy technologies, electrocatalysts are necessary to catalyze the critical chemical reactions, oxygen reduction reaction (ORR) and oxygen evolution reactions (OER) in fuel cells and metal–air batteries. Noble metals and their alloys (*e.g.*, Pt/C^2^ and RuO_2_/C^3^) are usually used as the catalysts in the energy devices. However, the noble metal catalysts are scarce and can lead to CO poisoning. To lower the cost and promote the commercialization of the clean energy technologies, extensive study has been made on a variety of materials including transition metals, metal oxides, carbon nanomaterials, and transition metal dichalcogenides. Some of these materials have exhibited high catalytic efficiency comparable to or better than the noble metal counterparts.^2–10^

Among the materials, Ag and its alloys are stable and only 2% as expensive as platinum. Experimental results have shown that Ag holds a great potential as a bifunctional catalyst for fuel cells and metal–air batteries especially in alkaline environments.^11–13^ In particular, Ag–Cu alloys or core–shell structures show better catalytic activities than the noble metals for ORR and OER. Nan *et al.* predicted from the first-principles calculations that the working potentials of pure Ag, core–shell Ag/Ag_3_Cu and alloy Ag_3_Cu were 0.737, 0.761 and 0.675 V, respectively, indicating that the core–shell Ag/Ag_3_Cu nanoparticles provide the highest working potential and the lowest overpotential, which is comparable to that of the Pt(111) facets.^14^ This prediction was confirmed by their experimental results. In these Ag-based structures, the transition metal Cu played an essential role in determining the electrochemical activities of the core–shell structures, similar to the alloys of platinum and early transition metals.^15–18^ Qaseem *et al.* summarized the silver core–shell structures for catalytic reactions such as Ag–Pd, Ag–Cu, Ag–Au and Ag–Co.^19^ Specifically, Ag–Pt catalyst exhibits three times better electrocatalytic performance than Pt.^20,21^ Simulation studies reveal that Ag_*x*_Au_*y*_ clusters enhance the adsorptions of CO and O_2_, and thus facilitate O_2_ dissociation and CO oxidation.^22^ Ag–Pt bimetallic nanoparticles are more stable than traditional Pt/C cathode and it can catalyze methanol oxidation.^23,24^ Shin *et al.* did DFT study on Ag–Pt cluster for comprehensive catalytic analysis.^25^ Strasser *et al.* found Pt-rich nanoparticles have compress strain resulting in a shift of band structure, thus improves the catalysis of reactions like ORR.^26^ Besides, Ag–Au cluster also shows higher catalytic activity than alloy and monometallic nanoparticles.^27,28^ Although the superior catalytic capabilities of the silver core–shell structures for ORR have been demonstrated theoretically and experimentally, the design principle for these structures is still underexplored. Establishing the design principle or descriptor that correlates core–shell structures to their catalytic activity will accelerate the search for highly efficient catalysts for clean energy conversion and storage.

In this paper, we performed the density functional theory calculations on various plh Ag_32_X_6_ and Cu_32_X_6_ core–shell metal structures (X = 3d transition metals) including Ag_32_Sc_6_, Ag_32_Ti_6_, Ag_32_V_6_, Ag_32_Cr_6_, Ag_32_Mn_6_, Ag_32_Fe_6_, Ag_32_Co_6_, Ag_32_Ni_6_ and Ag_32_Cu_6_. The overpotentials of OER and ORR were calculated in order to evaluate the electrochemical activities of the core–shell structures. Based on the DFT results, a design strategy was proposed to predict the catalytic performances of the core–shell metal clusters.

## Computational details

**Fig. 1:**
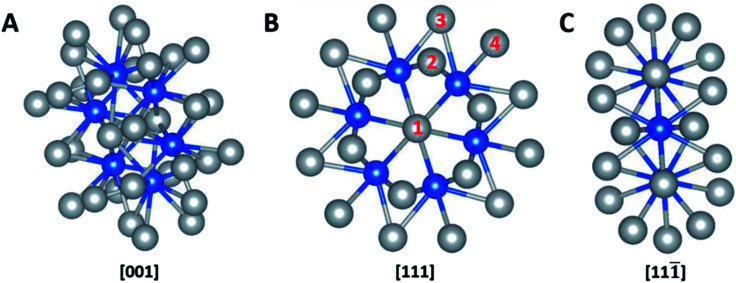
Schematic models of Ag_32_X_6_ plh core–shell structure. (A), [001] view; (B), [111] view; (C), [111̄] view. Four distinct active sites are marked in (B). Color legend: grey = shell elements, blue = core elements.

The computational calculation was performed by *ab initio* within the framework of the density functional theory (DFT) as implemented in Vienna Ab-initio Simulation Package (VASP). Ag_32_X_6_ polyicosahedral (plh) core–shell structures were constructed as shown in Fig. 1, in which X represents the 3d transitional metal (TM) elements (X = Sc, Ti, V, Cr, Mn, Fe, Co, Ni, Cu) in the periodic table. Ag atoms covered the X atom core to form the plh core–shell cluster. Zhang *et al.* showed that polyicosahedral (plh) Ag_32_Cu_6_ core–shell structure was more stable than truncated octahedral (TO) Ag_32_Cu_6_ one.^29^ In order to perform the DFT calculations, we identified 4 unique positions on particle surface as active sites that were marked by numbers in Fig. 1. The size of the unit cell is 20 Å × 20 Å × 20 Å with a bond length of 2.67 Å between Ag and X atoms approximately. For DFT simulations, projector augmented wave (PAW) pseudopotential was utilized to demonstrate the correlation of valence electrons.^30^ Generalized gradient approximation (GGA) was introduced by Perdew, Burke, and Ernzerhof (PBE) to state the electronic exchange and interactions.^31^ Brillouin zone sampling at the *Γ* point was used. An interatomic interaction in a Hartree–Fock like manner was used as the PBE+U method to correctly describe the systems with localized d and f electrons, typically transition metals.^19^*U* parameter was chosen based on previous calculations on bi-metallic structures.^32,33^ A cut-off energy of 480 eV was set throughout the calculations and the self-convergence level was set to be 1 × 10^−5^ eV for electron relaxations. Ionic relaxation converged when the total force reached less than 0.01 eV Å^−1^. Entropy and zero-point energy were also considered by vibrational frequency at 300 K. Bader charge analysis was conducted to evaluate the charge transfer within the nanoparticles.^34,35^

To carry out the calculations, two steps were conducted as lattice optimizations and electronic freedom re-optimized with no ions moved. After the Ag_32_X_6_ core–shell clusters were relaxed, we calculated the adsorption free energies of intermediates such as O, OH, O_2_ and OOH on the core–shell cluster to get free energies as well as overpotentials for ORR and OER. In this paper, both ORR and OER overpotentials were calculated to evaluate the bifunctionality of the structure for both reactions while the 4-electron transfer mechanism was believed to be the pathway for OER and ORR in fuel cells and metal–air batteries. The details regarding the overpotentials of OER, ORR can be found in various literatures such as Nørskov *et al.*,^2^ Man *et al.*,^3^ Zhao *et al.*,^5,36–39^ Li, *et al.*,^4^ and Zhang *et al.*^4,40–42^ Further details about the DFT calculations are provided in the ESI.†

## Results and discussion

The catalytic reactions were studied on plh core–shell metal clusters as shown in Fig. 1. Four distinct active sites on the surface of the core–shell structures are labeled in Fig. 1(B). The bridge sites are not taken into consideration in this study because previous work has shown that these bridge sites are not stable for adsorptions of intermediates.^29,43^ There are two major types of mechanisms towards OER and ORR: 4-electron (4e) transfer mechanism with the final production of H_2_O, and 2-electron (2e) transfer mechanism featuring the production of H_2_O_2_. The elementary steps of these 4e and 2e transfer reactions are shown in Fig. S1 and S2,† respectively.

**Fig. 2:**
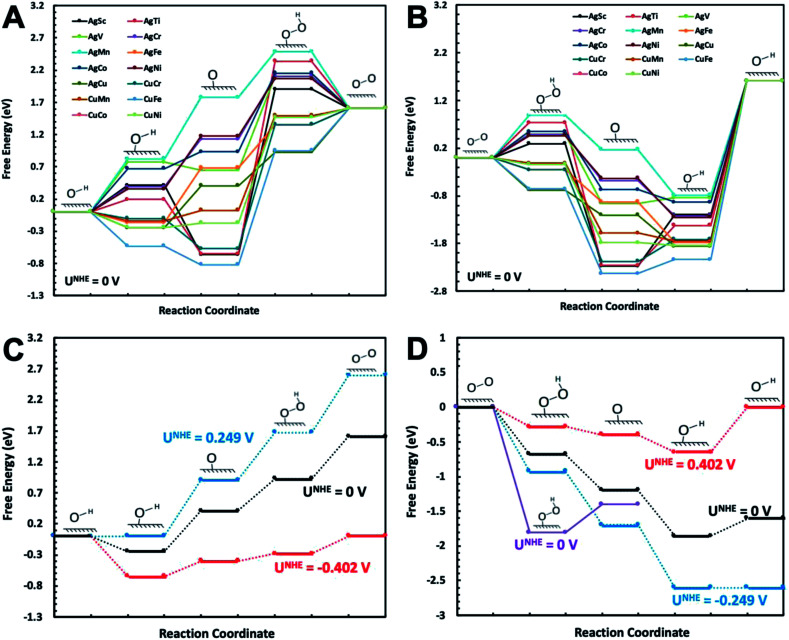
Free energy diagram of Ag_32_X_6_ and Cu_32_X_6_ plh core–shell structures at zero potential (*U*_0_ = 0 V) for (A) OER and (B) ORR in 4-electron transfer reaction mechanism in alkaline medium. Free energy diagram of Ag_32_Cu_6_ plh core–shell structure with the best catalytic performance at zero potential (*U*_0_ = 0 V), the equilibrium potential (*U*_0_ = 0.402 V), uphill/downhill potential (*U*_0_ = 0.249 V) for (C) OER and (D) ORR in alkaline medium.

Free energy diagrams of Ag_32_X_6_ and Cu_32_X_6_ structures for OER and ORR were plotted in Fig. 2(A) and (B), respectively. Among the core–shell structures studied in this study, Ag_32_Cu_6_ plh core–shell structure showed the best catalytic activities for OER and ORR since it has the smallest overpotential (the applied voltage that makes the reaction free energy in all elementary steps just uphill or downhill for OER and ORR, respectively). As shown in Fig. 2(C) and (D) the free energies for the Ag_32_Cu_6_ plh core–shell structure are plotted for the electrode potential of 0 V, 0.23 V and 0.402 V (equilibrium voltage for each step in OER and ORR). We also found sites 3 & 4 on the Ag_32_Cu_6_ plh core–shell structures (Fig. 1(B)) have better electrochemical activities of ORR and OER than sites 1 & 2 do. This is because sites 3 & 4 are outer-shell top sites while 1 & 2 are sort of bend inward, which may affect the adsorption of intermediates. The free energy diagram for 2-electron transfer mechanism was calculated and plotted in Fig. S3.† For comparison, the free energy of 2-electron transfer ORR mechanism of Ag_32_Cu_6_ plh core–shell structure was also plotted in Fig. 2(D). For the 2-electron transfer, the calculated electrode potential (overpotential) for Ag_32_Cu_6_ is 1.107 V, which was far larger than that for 4-electron transfer mechanism, indicating 2-electron mechanism is not favorable for plh core–shell metal structures.

**Fig. 3:**
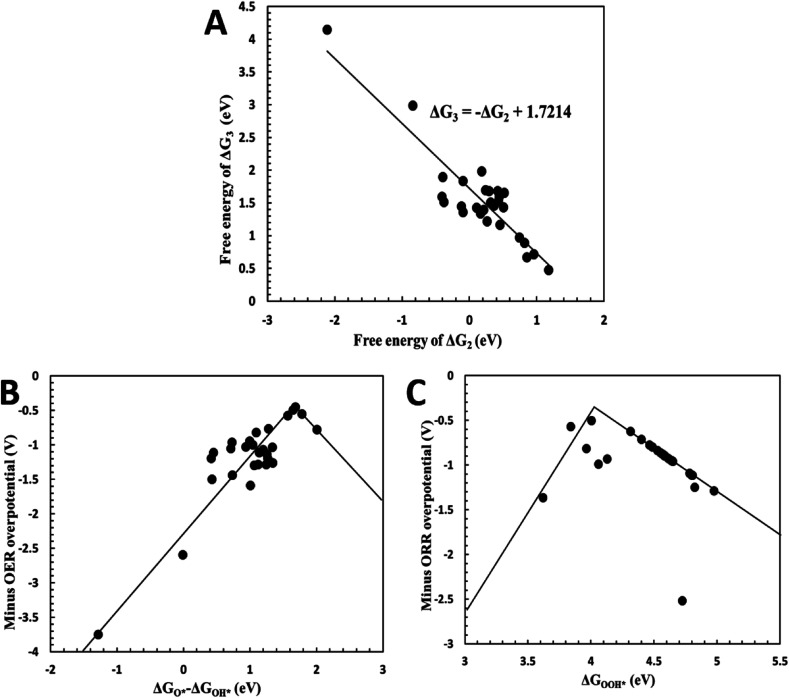
(A) Linear fitting of reaction free energy Δ*G*_3_ and Δ*G*_2_; (B) minus OER overpotential as a function of descriptor *G*_O^*^_ − Δ*G*_OH^*^_; (C) minus ORR overpotential as a function of descriptor Δ*G*_OOH^*^_.

Overpotential is an indicator of catalytic activities for electrocatalysts, which is the extra energy required for a reaction than thermodynamically expected. To theoretically evaluate the electrocatalytic performance of the plh core–shell structures, the adsorption free energies of O*, OH*, 
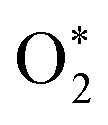
, OOH* (* refers to an active site on free surface) were calculated with the DFT method. Details regarding reaction equations, adsorption energies and overpotentials can be found in ESI.† We correlated the reaction free energy of OH* formation (Δ*G*_3_) with that of O* formation (Δ*G*_2_), as shown in Fig. 3(A). A linear regression was made with the linear least squares fitting technique and the fitting equation is Δ*G*_3_ = −Δ*G*_2_ + 1.7214. When Δ*G*_2_ equals Δ*G*_3_, the overpotential will approach its lower limit that is 0.459 V. This lower limit of the overpotential is comparable to Pt/C for ORR^2^ and RuO_2_/C for OER,^3^ as well as Pt-free catalysts for ORR or OER such as N-doped graphene,^9,10^ indicating Ag plh core–shell clusters can be used as an effective bifunctional catalyst for ORR and OER in fuel cells and metal–air batteries.

To establish design rules that correlate core–shell structures to their catalytic activity, the overpotentials was plotted as a function of the descriptors Δ*G*_O^*^_ − Δ*G*_OH^*^_ and Δ*G*_OOH^*^_. As shown in Fig. 3(B) and (C), OER overpotentials were drawn as a function of Δ*G*_O^*^_ − Δ*G*_OH^*^_ while ORR overpotentials were plotted against Δ*G*_OOH^*^_. Both of the plots show “volcano” relationships, on which the catalysts with the best performance are located at the summits of the volcanos. When *G*_O^*^_ − Δ*G*_OH^*^_ is around 1.6 eV, the OER overpotential achieves its lowest value (Fig. 3(B)), while the ORR overpotential has its lowest value at Δ*G*_OOH^*^_ = 4 eV (Fig. 3(C)). These volcano plots enable us to predict the best electrocatalytic properties of the catalysts by considering the reaction free energies only.

**Fig. 4:**
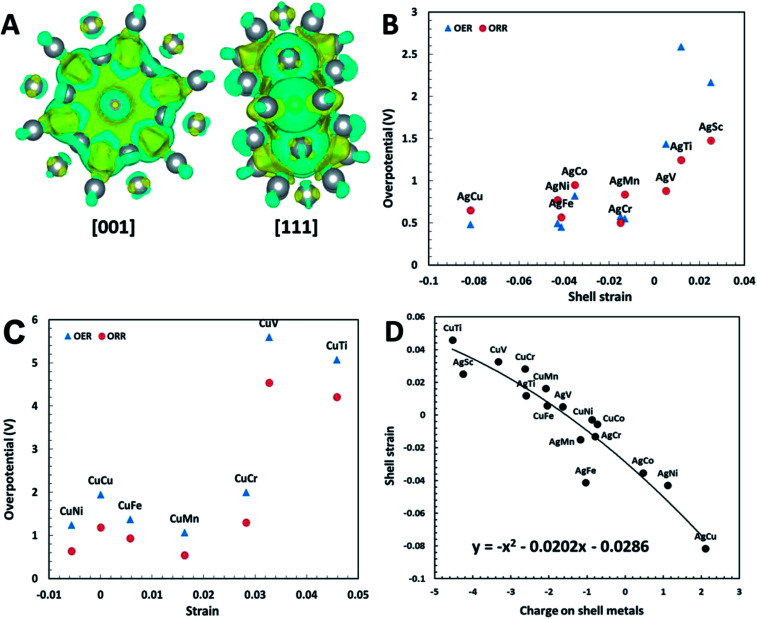
(A) Charge transfer of Ag_32_Cu_6_ plh core–shell structure. The blue and yellow colors indicate positive and negative charges, respectively. The isosurface level is set to be 0.001. (B) Relation between ORR/OER overpotentials and shell strain for Ag_32_X_6_ core–shell clusters; (C) relation between ORR/OER overpotential and shell strain for Cu_32_X_6_ core–shell clusters; (D) shell strain of core–shell structure as a function of charges on shell metals (Ag, Cu). The shell strain follows the equation of the 2^nd^ order polynomial trend line is *y* = −*x*^2^ + 0.0202*x* − 0.0286, where *x* is the charges on the shell.

Even though the descriptors Δ*G*_O^*^_ − Δ*G*_OH^*^_ and Δ*G*_OOH^*^_ can relate the overpotentials to the adsorption energies of intermediates, it is more desirable for a descriptor to directly correlate the core–shell structures to the activities. To relate the overpotentials to the intrinsic properties of the materials like what Zhao *et al.* did in their work.^37,38^ We use the strain on shell atoms of plh core–shell metal structure as a descriptor for OER and ORR. The shell strain is defined as,1
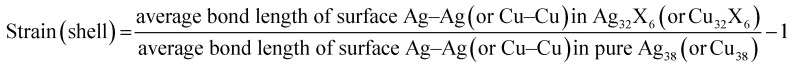
where the denominator is the average bond length of surface Ag–Ag in pure Ag_38_ metal clusters, and the numerator is the average bond length of surface Ag–Ag in the core–shell structures. This dimensionless factor successfully relates the activity to the intrinsic structural properties of Ag_32_X_6_ plh core–shell structures. We have plotted the overpotentials of each core–shell structure as a function of the shell strain. As can be seen from Fig. 4(B), the change of shell strain matches the sequence of the 3d transition metals in the periodic table. As the atom radius reduces from Sc to Cu, the shell strain reduces and transforms from tensile to compressive states, and consequently the overpotential becomes smaller successively. The Ag_32_Cu_6_ structure has identified to have the highest compressive strain, yielding the lowest overpotentials. Thus, the overpotentials or catalytic activities can be described well by the shell strain of the clusters.

To further verify the effectiveness of the shell strain in plh core–shell clusters as the descriptor for ORR and OER, we performed another set of calculations on Cu_32_X_6_ structures. These structures are comparable to the Ag_32_X_6_ clusters with the replace of Ag by Cu. We firstly relaxed the clusters, followed by the self-convergence computations without moving of ions. The Cu core–shell structure also has a unit cell of 20 Å × 20 Å × 20 Å and the bond length between Cu and X is about 2.47 Å. Since the Cu_32_Sc_6_ plh core–shell structure is unstable because of too large difference in radius between Cu and Sc, we excluded the analysis of Cu_32_Sc_6_. The free energy diagrams of Cu_32_X_6_ structures for 2e transfer mechanisms were also plotted in Fig. S4,† respectively, and the shell strain of Cu_32_X_6_ is also determined by eqn (1). As shown in Fig. 4(C), the overpotential for Cu_32_X_6_ structures reduces with reducing the shell strain, which follows the same trend of the Ag_32_X_6_ clusters. Thus, the shell strain is a general and intrinsic descriptor that is capable of predicting the catalytic activities of the plh core–shell bifunctional catalysts for OER and ORR, and could be extended to other core–shell alloys.

The excellent catalytic activities of the core–shell structures can be attributed to the charge transfer within the core–shell structures. It was shown for doped-graphene that the charge delocalization facilitated the electrocatalytic reaction.^4,40^ We have carried out Bader charge transfer analysis^34^ for Ag_32_Cu_6_ plh core–shell structure (Fig. 4(A)). After the formation of the core–shell structure, the charge transfer occurs on the surface. With the charge redistribution, the core (Cu) carries negative charge while the shell (Ag) becomes positively charged. The positively charged shell (Ag) can easily adsorb intermediates carrying negative charges, which is the essential in ORR and OER. According to Zhang *et al.*'s work, the O_2_ dissociation energy is 0.715 eV for plh Ag_32_Cu_6_ and the density of states (DOS) at the Fermi energy level is maximal for the favorable absorption site.^29^ Thus, the core–shell-induced charge transfer enhances the electrocatalytic activity of plh Ag_32_Cu_6_.

Similar Bader charge transfer analysis^34^ was also carried out for Cu_32_Ni_6_ structure with the lowest overpotential among all Cu_32_X_6_ clusters. As shown in Fig. S5,† the Cu shell has positive charges while the Ni core carries negative charges. Similar to Ag_32_Cu_6_ clusters, the positive charges on the shell of the whole structure is believed to enhance the electrochemical activity of the core–shell cluster. However, the amount of the charges transferred in Cu_32_X_6_ is lower than that in Ag_32_X_6_, indicating Ag_32_X_6_ will show better catalytic performance than Cu_32_X_6_. This conclusion is supported by the results in Fig. 4(B) and (C), in which the overpotentials of Cu_32_X_6_ are relatively larger than those of Ag_32_X_6_.

The shell strain of the core–shell cluster is also plotted as a function of charges on shell metals (Ag or Cu) for Ag_32_X_6_ and Cu_32_X_6_ structures in Fig. 4(D). As the charge on core metals increase, the shell strain also increase with a 2^nd^ order polynomial fitting of *y* = −*x*^2^ + 0.0202*x* − 0.0286 (round to *y* = −*x*^2^ + *x*). This demonstrates the charge transfer within the core–shell structures will influence the shell strain. Thus, one effective design strategy to enhance the electrocatalytic activities of plh core–shell metal clusters is to find the core–shell structures with the higher positive charge on shell metals.

## Conclusions

Ag_32_X_6_ and Cu_32_X_6_ plh core–shell structures and their electrocatalytic properties were studied systematically with the DFT methods. The Gibbs free energy, overpotential of OER and ORR on the core–shell structures were calculated to evaluate the electrocatalytic performances. Our results show that Ag_32_Cu_6_ plh core–shell metal clusters exhibit the lowest overpotential comparable to Pt. The shell strain is found to well describe the catalytic activities of the core–shell structures. Increasing the compressive shell strain will induce more positive charge on the shell of the core–shell structures, thus enhancing the adsorption and subsequent reactions on the structures. The design principles developed from the intrinsic descriptor enables the rational design and screening of the core–shell structures for high-performance catalysts by evaluating the strain on the shell.

## Conflicts of interest

There are no conflicts to declare.

## Supplementary Material

RA-009-C8RA09122F-s001
