# Supplementary material for: Rational design of efficient transition metal core–shell electrocatalysts for oxygen reduction and evolution reactions
Source: RSC Adv. 2019 Jan 2;9(1):536–42. doi: 10.1039/c8ra09122f (PMC9059333; doi:10.1039/c8ra09122f)
Supplement: RA-009-C8RA09122F-s001 [file RA-009-C8RA09122F-s001.pdf]

Electronic Supplementary Material (ESI) for RSC Advances.
This journal is © The Royal Society of Chemistry 2018

Supplementary Information

Rational Design of Efficient Transition Metal Core-Shell Electrocatalysts for Oxygen Reduction and Evolution Reactions

Zhenghang Zhao[1], Jason D'Souza[1], Fuyi Chen[2], Zhenhai Xia[1,2,]*

1 Department of Materials Science and Engineering, University of North Texas, Denton, TX, 76201, USA

2, School of Materials Science and Engineering, Northwestern Polytechnical University, Xi'an 710072, China

*Corresponding author: Zhenhai.Xia@unt.edu

1. Reaction pathways of OER and ORR

For oxygen reduction reaction (ORR), there are mainly two reaction mechanisms, 4-electron transfer mechanism in which O_2 is reduced into H_2O and 2-electron transfer mechanism in which O_2 is reduced into H_2O_2. Both of these two mechanisms could occur in fuel cells and metal-air batteries. In our study on core-shell metal clusters, both reactions are considered. For 4-electron transfer mechanism, ORR in an alkaline environment occurs following these elementary steps,

$$O_2 + * \rightarrow O_2^* \tag{S1}$$

$$O_2^* + H_2O(l) + e^- \rightarrow HOO^* + OH^- \tag{S2}$$

$$HOO^* + e^- \rightarrow O^* + OH^- \tag{S3}$$

$$O^* + H_2O(l) + e^- \rightarrow HO^* + OH^- \tag{S4}$$

$$HO^* + e^- \rightarrow OH^- + * \tag{S5}$$

The oxygen evolution reaction (OER) is opposite to ORR in an alkaline environment, and can be written as,

$$OH^- + \ * \rightarrow HO^* + e^- \tag{S6}$$

$$HO^* + OH^- \rightarrow O^* + H_2O(l) + e^- \tag{S7}$$

$$O^* + OH^- \rightarrow HOO^* + e^- \tag{S8}$$

$$HOO^* + OH^- \rightarrow O_2^* + H_2O(l) + e^- \tag{S9}$$

$$O_2^* \rightarrow O_2(g) + \ * \tag{S0}$$

The ORR could also follow 2-electron transfer pathway, as follows.

$$* + O_2 = O_2^* \tag{S11}$$

$$O_2^* + H_2O(l) + e^- \rightarrow HOO^* + OH^- \tag{S12}$$

$$OOH^* + H_2O(l) + e^- = H_2O_2 + OH^- + \ * \tag{S13}$$

In this two-electron transfer ORR, instead of producing a water molecule, it produces a hydrogen peroxide molecule.

The Gibbs free energy of each step in both of those two mechanisms can be calculated by

$$\Delta G = \Delta E + \Delta ZPE - T\Delta S + \Delta G_U + \Delta G_{pH} \tag{S14}$$

where ΔE is the reaction free energy obtained from the DFT simulations, ΔZPE is the difference of zero-point energy before and after the reaction obtained from Density Functional Perturbation Theory (DFPT) calculations, ΔS is obtained by vibrational frequency at 300K, $\Delta G_u = -eU$, e is electron, U is the potential on the electrode and ΔG_{pH} is the correction for pH value and can be further described as,

$$\Delta G_{pH} = -k_B T \ln[\mathrm{H}^+] \tag{S15}$$

where k_B is the Boltzmann constant. The overpotential of OER for 4-electron transfer mechanism is listed as below,

$$G^{OER} = \max\{\Delta G_1, \Delta G_2, \Delta G_3, \Delta G_4\} \tag{S16}$$

$$\eta^{OER} = \frac{G^{OER}}{e} - 0.402\ V \tag{S17}$$

In contrast, the overpotential of ORR for 4-electron transfer mechanism is listed as below,

$$G^{ORR} = \min\{\Delta G_1, \Delta G_2, \Delta G_3, \Delta G_4\} \tag{S18}$$

$$\eta^{ORR} = |G^{ORR}|/e - 0.402 \tag{S19}$$

For 2-electron transfer mechanism shown in Eq. S11-S13, the overpotential is simply the adsorption free energy of OOH* minus the equilibrium potential 0.7 V, because there are only two electrons transferred in the reactions. However, in alkaline media, there is still a correction for the pH value as shown in Eq. S15. The equation is written as below,

$$\Delta G_{OOH*} = G_{OOH*} - G_{O^2} - G_{*} - \frac{1}{2}G_{H^2} \tag{S20}$$

$$\eta = \Delta G_{OOH*} + 0.104 \tag{S21}$$

2. Figures and Tables

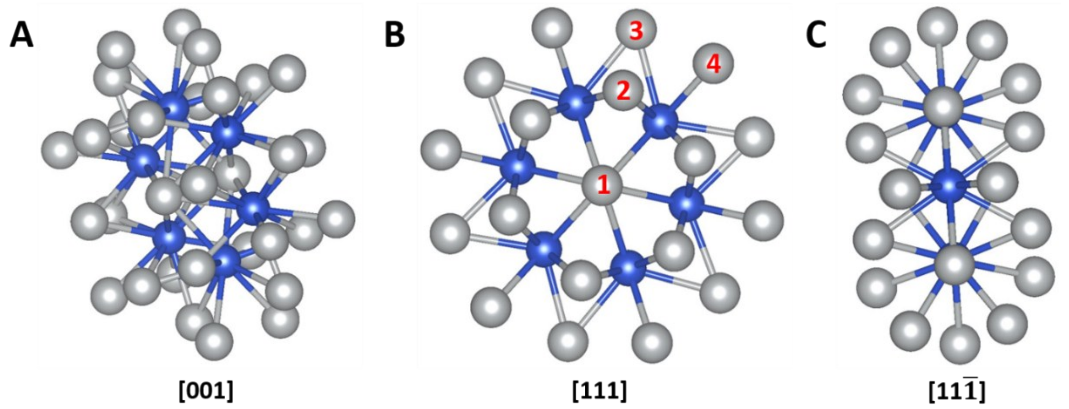

Figure S1. Schematic models of $Ag_{32}X_6$ plh core-shell structure. (A), [001] view; (B), [111] view; (C), [11$\bar{1}$] view. Color legend: Large grey=shell elements, blue=core elements.

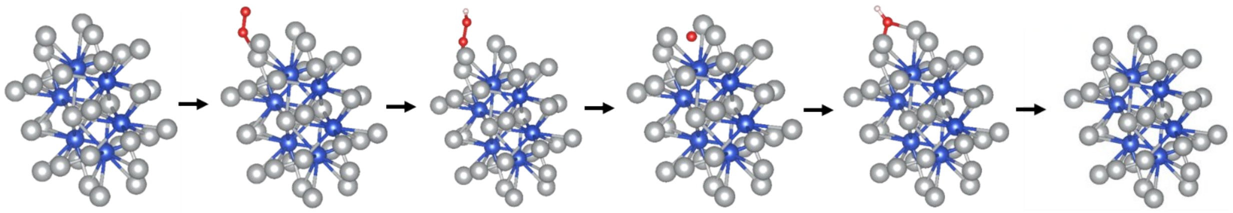

Figure S2. Whole process of ORR on plh core-shell metal structure for 4-electron transfer mechanism. The reversed process is for OER. Color legend: Large grey=shell elements, blue=core elements, red=oxygen and small grey = hydrogen.

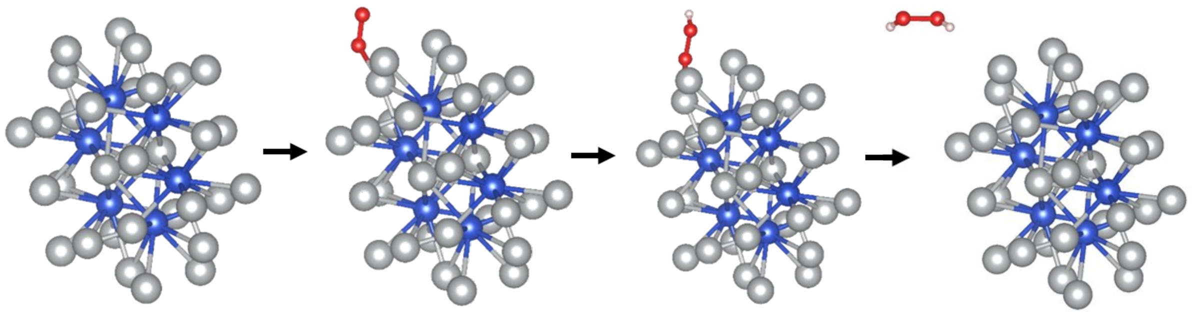

Figure S3. Whole process of ORR on plh core-shell metal structure for 2 electron transfer mechanism. Color legend: Large grey=shell elements, blue=core elements, red=oxygen and small grey = hydrogen.

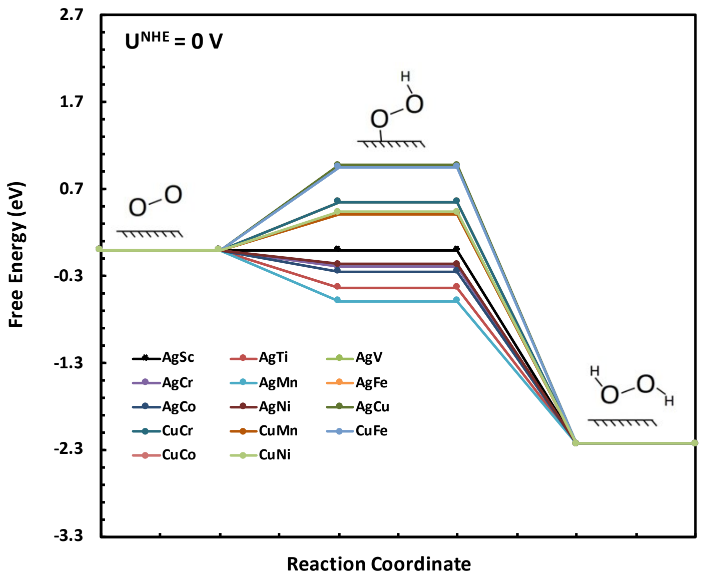

Figure S4. Free energy diagrams of $Ag_{32}X_6$ and $Cu_{32}X_6$ plh core-shell structure with the best catalytic performance at zero potential (U0= 0 V) for 2 electron ORR in alkaline medium.

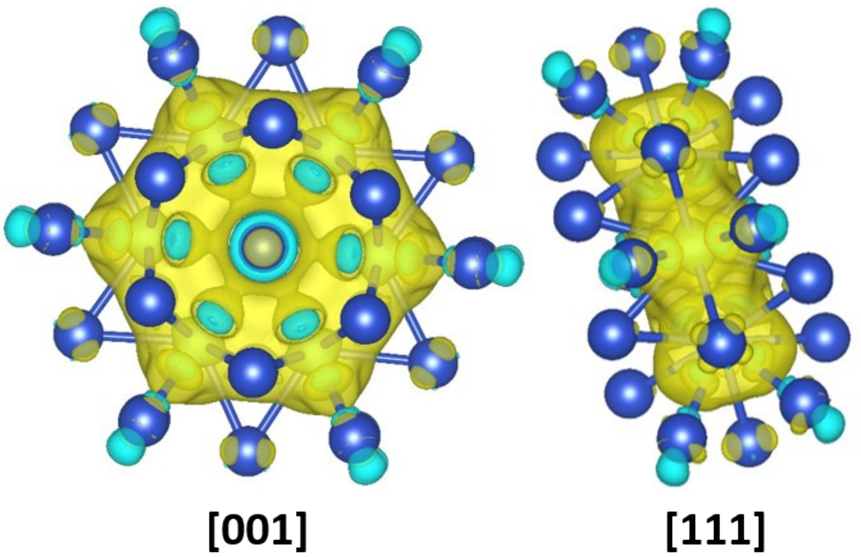

Figure S5. Charge transfer within $Cu_{32}Ni_6$ plh core-shell structure. The blue color indicates positive charge and the yellow color indicates negative charge. The isosurface level is set to be 0.001.
